# Learning intraoperative organ manipulation with context-based reinforcement learning

**DOI:** 10.1007/s11548-022-02630-2

**Published:** 2022-05-03

**Authors:** Claudia D’Ettorre, Silvia Zirino, Neri Niccolò Dei, Agostino Stilli, Elena De Momi, Danail Stoyanov

**Affiliations:** 1 Wellcome/EPSRC Centre for International and Surgical Sciences (WEISS), University College London, London, UK; 2 Department of Electronics, Information and Bioengineering (NearLab), Politecnico of Milan, Milan, Italy; 3 The BioRobotics Institute, Scuola Superiore Sant’Anna, Pisa, Italy

**Keywords:** Computer-assisted intervention, Robotic surgery, Reinforcement learning, Surgical automation

## Abstract

**Purpose:**

Automation of sub-tasks during robotic surgery is challenging due to the high variability of the surgical scenes intra- and inter-patients. For example, the pick and place task can be executed different times during the same operation and for distinct purposes. Hence, designing automation solutions that can generalise a skill over different contexts becomes hard. All the experiments are conducted using the Pneumatic Attachable Flexible (PAF) rail, a novel surgical tool designed for robotic-assisted intraoperative organ manipulation.

**Methods:**

We build upon previous open-source surgical Reinforcement Learning (RL) training environment to develop a new RL framework for manipulation skills, *rlman*. In *rlman*, contextual RL agents are trained to solve different aspects of the pick and place task using the PAF rail system. *rlman* is implemented to support both low- and high-dimensional state information to solve surgical sub-tasks in a simulation environment.

**Results:**

We use *rlman* to train state of the art RL agents to solve four different surgical sub-tasks involving manipulation skills using the PAF rail. We compare the results with state-of-the-art benchmarks found in the literature. We evaluate the ability of the agent to be able to generalise over different aspects of the targeted surgical environment.

**Conclusion:**

We have shown that the *rlman* framework can support the training of different RL algorithms for solving surgical sub-task, analysing the importance of context information for generalisation capabilities. We are aiming to deploy the trained policy on the real da Vinci using the dVRK and show that the generalisation of the trained policy can be transferred to the real world.

**Supplementary Information:**

The online version contains supplementary material available at 10.1007/s11548-022-02630-2.

## Introduction

**Fig. 1:**
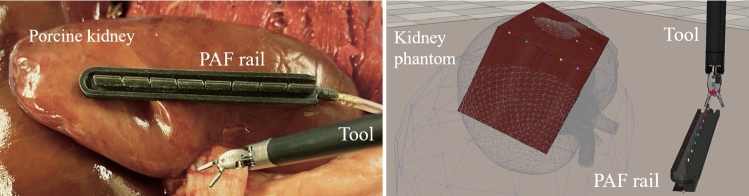
Representation of an example surgical scene for organ repositioning. The PAF rail and the robotic tool are also represented in *rlman* environment

**Table 1:** Overview of surgical robotics training environments

	Softwares	Physics	States	Actions	Tasks	Bimanual	ECM	Objects
dVRL	Python, V-REP	Static+	Low dim	3	2	No	No	Cylinder, target
UnityFlexML	Python, Unity	Static+	Low dim	3	2	No	No	Tissues
SurRoL	Python	Dynamic	Low, high dim	4	2	Yes	Yes	Needle, Blocks, etc.
DeformerNet	Isaac gym	Dynamic	Low, high dim	4	2	No	Yes	Tissues
rlman	Python, CoppeliaSim	Static+	Low, high dim	4	2	No	Yes	PAF rail, Kidney, etc

Static+: simplified grasping strategy, with limited physical interaction

The adoption of Robotic Minimally Invasive Surgery (RMIS) has been growing worldwide thanks to the introduction of robotic platforms like the da Vinci Surgical System (Intuitive Surgical Inc., Sunnyvale, CA, US). Besides its extensive clinical use, the da Vinci System has been widely investigated by researchers thanks to the open-source da Vinci Research Kit (dVRK) [1] developed by Johns Hopkins University in the early 2010’s. The dVRK enabled the development of a vibrant research community that has been investigating different robotic surgery problems [2]. With recent advances in machine learning, learning-based approaches for robotic surgery task understanding and automation, have become and active area of research [2]. Among these approaches, researchers have shown how with Reinforcement Learning (RL) it is possible to solve tasks in different environmental conditions without the need of a human to tailor the algorithm to specific solutions once conditions change. This work builds upon a previously developed open-source RL-oriented and dVRK-compatible simulation environment [3] to develop *rlman*, a framework for RL training of manipulation skills. The *rlman* framework can support diverse surgical operation scenarios. To demonstrate its capabilities, we present a case study focused on the use of the PAF rail system for intraoperative organ repositioning [4]. Code is publicly available at https://github.com/Cladett/rlman. The paper’s main contributions can be summarised as follows:

- We build upon the previous dVRL work [3] to develop *rlman*, a training framework for manipulation skills.
- We conduct extensive experiments on RL algorithms comparing four different manipulation skills with state-of-the-art benchmarks [5].
- We implement contextual RL agents which are able to generalise over context meta data.
- We evaluate the ability of the trained agent to generalise over the pick and place task environment conditions.

## Related work

### Automation of intra-operative organ manipulation

During RAMIS, surgeons frequently reposition tissues and organs with the help of the tool shafts. The PAF rail, shown in Fig. 1, can be of great help for the surgeon when performing the aforementioned procedures [6, 7]. The PAF rail uses a series of vacuum-actuated suction cups to pair with the targeted organ/tissue providing a flexible interface that enables safe manipulation. Given the PAF rail design, the clinical accuracy for this application can be measured as the orientation of the rail compared to the organ surface. To guarantee a good suction, able to hold the weight of the organ, the rail base needs to be parallel to the tissue. If this condition is not verified, when the suction pump is turned on, the rail is not able to guarantee a perfect suction with the organ tissue and a loss of pressure occurs. More details are provided in the supplementary material. Automating this step would allow the surgeon to keep active control over the two main arms, saving precious operating time without repeatedly switching to the third arm.

### RL in surgical robotics

Reinforcement Learning (RL) is a machine learning training method [8] that has been used vastly in robotic industrial applications [9]. Because of the RL paradigm, to successfully train the agent, it needs to interact with the environment hundreds of thousands to millions of times, making it fundamental to have an efficient and lightweight simulation environment. The Surgical Robotics learning simulation environments currently available are presented in Table 1. Before these learning environments were introduced, two main simulation environments were openly released in [10], and [11], empowering the following developments. In the former, the authors released the first V-REP model of the da Vinci, and in the latter some learning support was introduced. The first work that bridges the gap between medical robotics and RL is the dVRL presented in [3]. This work introduces the first learning environment compatible with OpenAI *Gym* and the dVRK. Other works focused on environments with better dynamics interactions between the da Vinci tools and soft tissues [12, 13]. These works have been followed by more recent works presented in [5, 14] where there has been improvement in terms of environment dynamics and task characterisation.

## Methods

### Goal oriented RL

Reinforcement Learning is a paradigm used to solve problems that can be formalised as an agent interacting with an environment. Formally, this type of problems is known as Markov Decision Process (MDP). An MDP is defined by the following tuple: $$ \langle S, A, P, \gamma , R\rangle $$, where *S* represents the state space for which a given state $$s_{t} \in S$$, *A* the action space for which $$a_{t} \in A$$, *P* the dynamics probabilities $$ p(s_{t+1}$$ |$$s_t, a_t)$$, and $$\gamma $$ is the discount factor and *R* the reward space for $$r_t \in R$$. The aim of RL is to optimise a policy $$\pi $$ such as $$a_{t} = \pi _{\theta }(s_{t})$$ to maximise the expected return $$\mathbb {E}[\sum _{t=0}^{H} \gamma ^{t} r_{t}]$$, where *H* represents the horizon [8]. In goal-oriented RL algorithms, the aim is to learn a parametrised Q-function, defined as $$Q_{w} (s, a, g)$$, that estimates the expected return of taking an action *a* from state *s* with goal *g* and the parameters *w*. In Q-learning problems, the solution relies on the minimisation of the Bellman [8] error:

1 $$\begin{aligned} \mathcal {L} = Q_{w}(s, a, g) - (r + \max _{a'}Q_{w}(s', a', g)) \end{aligned}$$

Standard actor-critic algorithms can be used to optimise this objective, using a set of transitions $(s, a, s', g, r)$ which can be collected off-policy. In practice, a target network is often used for the second Q-function.

**Fig. 2:**
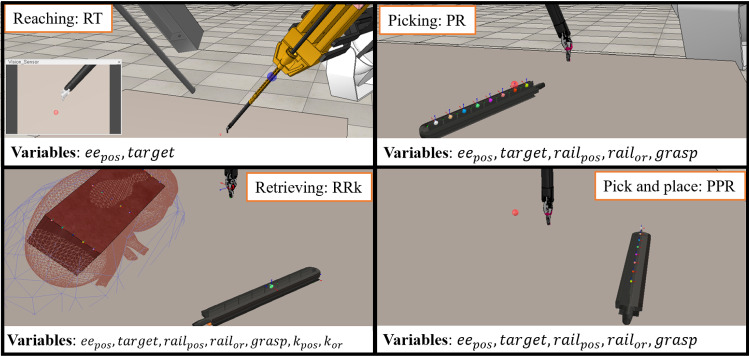
Representation of the four environments used to train the different skills and their respective environment variables. Starting from the top left clockwise: reaching skill characterised by the ReachTarget (RT) environment, shown here, and ReachRail (RR), picking skill with the PickRail (PR) environment, the pick and place with PickPlaceRail (PPR) and retrieving skill with RetrieveRailkidney (RRk)

### Contextual RL

Manipulation tasks can be defined as a sequence of different steps, executed one after the other applying different skills. For example, the pick and place task can be divided into different surgemes each of which can be associated with different skills. Therefore, autonomous agents need to master multiple skills in a sample-efficient manner to adapt effectively to real world scenarios. Multi-Task Reinforcement Learning (MTRL) represents a promising approach to train effective real-world agents [15]. As previously described for goal oriented RL, contextual RL aims to solve problems known as Contextual MDP (CMDP) [16]. The first proposed version of CMDP in [17] defines context as augmentation of MDP using side information as form of context, in a similar fashion to contextual bandits. Contextual MDP (CMDP) can be defined as follows:

#### Definition 1

A CMDP is defined by a tuple $\langle C, S, A, M \rangle$ where *C* is the context space, *S* is the state space and *A* is the action space. *M* is a function that maps a context $c \in C$ to MDP parameters $M(c) = \{R^{c}, T^{c}\}.$

In a multi-task setting, where a family of MDPs is defined as each MDP with a shared state space *S*, context can be applied. However, $S^{c}$ (either low-dimensional or high, like pixels) is the only state space the agent has access to, and it represents a subspace of the original state space S, focusing only on objects relevant to the task analysed. Different MDPs can be characterise by different combinations of objects and skills. Therefore, the state space $S^{c}$ and reward function $R^{c}$ can differ across MDPs as well as the $T^{c}$ representing the transitions probability function. However, when objects are shared across tasks, their dynamics remain consistent across them. In this work, we defined $S^{c}$ as the state space of an environment characterised by obstacles with different shapes and dimensions that can vary among pre-defined sets of values. We measure the performance of RL methods in a range of unseen (yet related) environments with different environment configuration.

### *rlman* library

*rlman* trains different parts of the intraoperative organ manipulation task (Fig. 2), inside the same environment space. Following the architecture introduced in [3], all the environments can interface in a *Gym*-like fashion with state-of-the-art RL libraries.

#### Environment variables

**Fig. 3:**
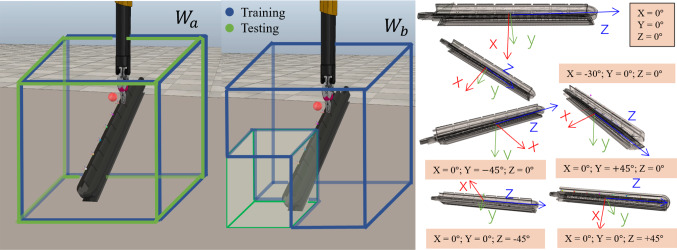
Definition of the workspace volumes on the left, and of the orientation randomisation ranges for the rail on the right

The training of each skill takes place in a different environment. We have defined a set of environment variables that are randomised at every reset of an episode for each environment. The variables have been selected to make the experiment as similar as possible to the actual clinical scenario. For the reach skill, the variables randomised are the initial tooltip position, the target position, the initial position and orientation of the rail along with the grasping site. Lastly, the retrieving environment also has the randomisation of the initial position and orientation of the kidney with the target location belonging to the kidney surface. We define the orientation range for the rail based on potential configurations in a real clinical scenario, see Fig. 3, right side.

*Workspace volumes* We defined the randomisation of the workspace for the scene object as a cubic volume bounded by $\omega \in \mathbb {R}^+$ and centered around the position ${\varvec{p}}_{{\varvec{home}}} \in \mathbb {R}^3$. The entire workspace $W_a$ can then be defined as:

2 $$\begin{aligned} W_a= & {} \{ \mathbf{p} \nonumber \\= & {} [p_x, p_y, p_z] \in \mathbb {R}^3 : \forall x \in \varvec{p_{home}} - \omega< x < \varvec{p_{home}} + \omega \}\nonumber \\ \end{aligned}$$

Moreover, for the experiment described in section “Generalisation over context meta data” we proposed a different formulation for the workspace as $W_{b} = W_{train} + W_{test}$, with $W_{test}$ defined as one eighth of the full volume $W_b$ and $W_{train}$ the remaining portion, as shown in Fig. 3.

#### Action space

The RL agent is responsible for selecting the next Cartesian position of the robot as well as the gripper orientation, and the inverse kinematics is then computed inside the environment [10]. The jaw angle $j_t$ is ranged between [0, 1], with 0 being completely closed and 1 entirely open. As shown in Table 1, the grasping is characterised by the following simplified strategy: objects are considered grasped when $j_t < 0.25$ and the proximity sensor located in between the tooltip grippers is triggered. As suggested in [18], actions are normalised in the following way:

3 $$\begin{aligned} p_{t+1}= & {} \sigma \Delta _t + p_t \end{aligned}$$

4 $$\begin{aligned} j_{t+1}= & {} (\psi _t + 1) / 2 \end{aligned}$$

where $\Delta _t, \psi _t \in [-1, 1]$ represent the actions that the agent can choose. $\sigma$ represents a safety threshold that restricts the maximum step size between different actions.

#### State space

*rlman* supports both low-dimensional Cartesian coordinates and high-dimensional states represented by RGB mask images rendered by OpenGL. In the first case, the difference in complexity of the state space allows the agent to focus on continuous control learning with sample efficiency while sacrificing the perception of the task. In the second case, image perception, which represents powerful control data in robotics, is required. All the experiments have been conducted with low-dimensional state space definition, apart from the reach skill environment where high-dimensional state spaces have been investigated. For the low dimension case, as for the actions, the values are randomised based on $\hat{p_t} = (p_t - p_{home}) / \omega$. For the reach skill environment, the images are acquired by a single vision sensor attached to the Endoscope Camera Manipulator (ECM). The frames are first converted from RGB to grey-scale and then down-sampled from 320x240 to 64x64 in order to decrease the computational load.

#### Reward functions

Reward shaping is a crucial step when designing a RL problem [19]. In *rlman* we aim to investigate the agents’ capability of generalising. Given a success function *h*(*s*, *g*), where *g* represents the designed goal for the task, the reward will be 0 when the task is successful and $-1$ otherwise. As a result, binary rewards can be defined as follows:

5 $$\begin{aligned} r(s,g) = {\left\{ \begin{array}{ll} 0 &{} \text {if } h(s,g) > \partial \\ -1&{} \text {if } h(s,g) < \partial \end{array}\right. }\, \end{aligned}$$

where $\partial$ represents the acceptance threshold. Details on the success function will be provided on the experiment section. Given the complexity of the task, for the reach skill in the high-dimensional case we opted for the following dense reward $r(s) = - k(\omega ) \Vert h(s, g)\Vert$, where *k* is the normalisation factor.

#### Algorithms

We evaluate every skills environment with model-free RL algorithms, including the on-policy method Proximal Policy Optimisation (PPO) [20], the off-policy method Deep Deterministic Policy Gradient (DDPG) [21] also tested with Hindsight Experience Reply (HER) [22], as described in section “Goal oriented RL”. For the high-dimensional environment, we also used Deep Q-Networks (DQN) [23].

## Experiments

### *rlman*-skills evaluation

We divided the intraoperative organ manipulation task into different part that we associated with a different skill and trained them in the *rlman* framework. These skills were selected based on the need of dexterity and high precision in the surgical context of intraoperative organ manipulation applied to the PAF rail test case. All the experiments were run using as robotic tool the Large Needle Driver (LND). For the experiments, the manipulation workspace was set to $\omega = 5$ *cm* and the success function set to $h(s,g) = \omega \Vert p_t - g \Vert < \partial$, where $\partial$ is set to 3 mm.

1. *Reach Rail* this environment was mainly used as validation for testing the feasibility of using low-dimensional state information (RR - ReachRail) and high-dimensional ones (RT - ReachTarget). In both cases, the goal is to reach a circular target whose position is randomised, without considering jaw angles. For the RR case, the tooltip position has to reach the target over the rail within a tolerance and to be perpendicular to the table’s surface. For RT, the relative position between the endoscope and the robotic arm is selected based on [24], to maximise the camera field of view.
2. *Pick Rail* in this environment (PR) we introduce the jaw angle. The robotic tool has to approach the rail and actually open and close the jaw to ensure a correct grasp. The PAF rail is characterised by eight different grasping sites, which are randomised during training.
3. *Retrieve Rail* this skill aims to train the capability of properly locating the rail over the target organ. Fundamental task to assess the clinical accuracy, the base of the rail needs to be parallel to the kidney surface. The RetrieveRailkidney (RRk) is characterised by a kidney phantom representing the target. Further details on the phantom choice are provided in the supplementary material.
  The goal of this task is to retrieve the rail, whose starting configuration is fixed, and place it over the kidney target position with the correct orientation to guarantee a successful suction.
4. *Pick and place Rail* in this environment (PickPlaceRail, PPR) the goal is to sequentially pick the rail and drive it towards the target position. Grasping site, rail position and target are all randomised inside the training volume.

Due to the extreme challenge of learning manipulation skills that need to be transferable to a surgical environment characterised by small size objects and high-precision movements, we decided to adopt low-dimensional state representation when involving the rail, and test the high-dimensional one as proof of concept using a simplified circular target.

**Fig. 4:**
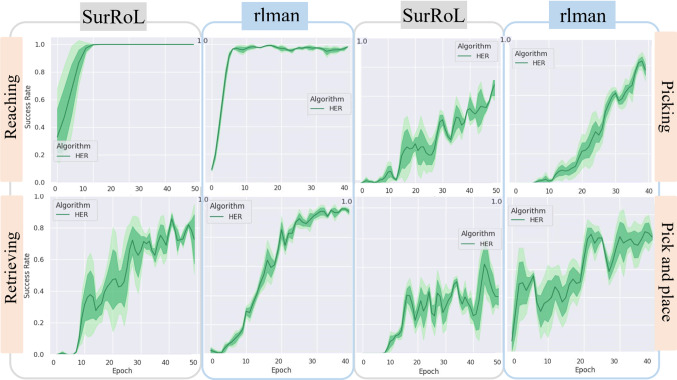
Average success rate during testing of the analysed skills for each environment shown over four random seeds, with one epoch set as 40 episodes. The average value are represented by continuous line, the lighted shaded region represent the standard deviation (std); and the dark shaded region the standard error. A more detail version can be found in the supplementary material

### Generalisation over environment variables

We selected the PPR environment to run further experiments to assess the capability of the agent to generalise over environment variables and unseen portions of the training volumes.

**Table 2:** Randomisation of the environment variables in PPR

	*Grasp*	$rail_{pos}$	$rail_{or}$	$ee_{pos}$	*Target*
E1	rnd	Fixed	Fixed	Fixed	Fixed
E2	rnd	rnd	Fixed	Fixed	Fixed
E3	rnd	rnd	rnd	Fixed	Fxed
E4	rnd	rnd	Fixed	Fixed	rnd
E5	rnd	rnd	rnd	rnd	Fixed
E6	rnd	rnd	rnd	rnd	rnd

As highlighted in Table 2, we defined the five different environment variables described in section “*rlman* library” as: $$ grasp_{site}$$, $$rail_{pos}$$, $$rail_{or}$$, $$ee_{pos}$$ and *target*. We ran six different experiments, indicated by the **E#**, randomising, for each experiment, different sets of environment variables. In addition, each of these experiments have been run twice in order to test them using the two different volumes $$W_a$$ and $$W_b$$ defined in section “*rlman* library”.

### Generalisation over context meta data

We augmented the PPR environment with three additional obstacles ideally representing anatomical regions we want the RL agent to avoid when executing the pick and place task. A picture of the environment is reported in the supplementary material. We wanted to test if the agent could execute the task successfully regardless of the condition of the context of the experiments. Therefore, obstacles have been added to the environment to simulate anatomical regions. Whenever a collision is detected between the robot, the PAF rail and any of the obstacles, the environment resets and the episode restarts. This environment behaviour will discourage the agent to move close to the obstacles, learning behaviour that can translate safely for future clinical practice. As explained in section “*rlman*-skills evaluation”, the anatomy was approximated with a cuboid shape to reduce overhead. As described in the methods section, we enrich the state space using the meta data characterising these obstacles. Specifically, we input the 3D Cartesian position of the centroid of each cube and their size (represented by their volumes). The size is randomised at every episode with the following criteria: given $\delta _l = 2 mm$ defined as the incremental factor, each side of the cuboids at every reset can be randomised inside the following interval $[l_{0} \pm \delta _l]$, where $l_0$ is the size of each side chosen as reference for each cuboid.

**Fig. 5:**
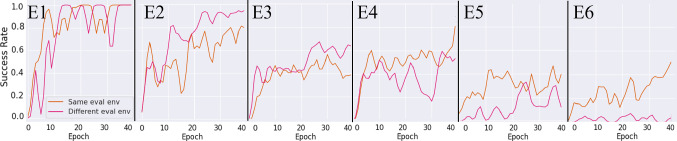
Representation of the results during testing for the PPR environment. Number of experiments refer to Table 2

**Fig. 6:**
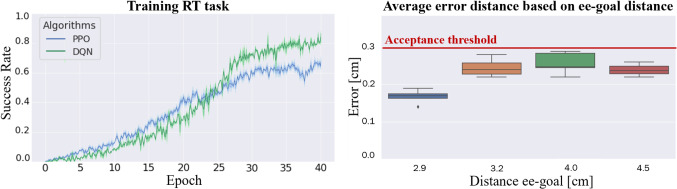
On the left: success rate for RT task. On the right: error representation between final tool position and goal compared to their initial distances

## Results

### Skills benchmarks

It becomes very challenging for a RL agent to solve multi-step tasks, such as pick and place, without any previous knowledge of the task [19]. Therefore, for the PR and PPT tasks we integrated demonstrations into the training process, as done in [25], by collecting 50 episodes for each of the tasks. We heuristically control the robot in simulation to record the demonstrations. Figure 4 shows a summary of the results of the evaluation for RL agents. We use the success rates during testing as evaluation metric for the results of the experiments [5]. For representation purposes, we reported only the algorithm showing positive results, for complete picture refer to the supplementary material. The results for training agents in *rlman* are compared to *SurRoL* benchmarks. For the comparison, we selected only the SurRoL environments that were presenting the same state-space definition adopted in *rlman*. Therefore, even if the task had slightly different definition, e.g. using a different object, the characterization of the problem remains the same. We replicate the results on our machine only for tasks that are strictly related to the skills analysed in *rlman*. Each experiment was run four times, randomly changing the seed for every execution. The supplementary material provides a more comprehensive picture showing also the training graphs.

### Pick and place results

The results from the pick and place generalisation experiments are shown in Fig. 5. For full training and testing results, refer to the supplementary material. As it is possible to notice, increasing the number of randomised environment variables negatively affects the agents’ performance, especially when randomising $rail_{or}$ and $ee_{pos}$. When the rail orientation is not randomised, the rail is always sitting over the table surface. When we introduce random rail orientation, the rail’s position is shifted in order to avoid any penetration with the table’s surface. Hence, the possible configuration space of the rail becomes much bigger and with more complex entries, affecting the agent’s performance. When randomising $ee_{pos}$, it could happen that the relative distance between robotic tool, rail and target is too long for the agent to be able to solve the task within the maximum number of time steps declared for that task, see supplementary material for example. This is not the case when there is no randomisation of $ee_{pos}$ because the volumes are always defined around ${\varvec{p}}_{{\varvec{home}}}$, as described in section “*rlman* library”.

## Discussion and conclusion

We presented *rlman*, a learning framework for manipulation skills compatible with the dVRK. Four learning-based surgical manipulation skills were developed and validated using the PAF rail system as a test case. Extensive experiments were carried out in simulation to compare skills trained in *rlman* with similar ones trained in the benchmark environment. Despite the tasks present the same formulation, differences encountered in the results might be due to different environment’s formulation and variables. We also reported results, Fig. 6, of using single camera frames as state information and how the agent can learn how to solve the task using this information. Further experiments were developed in the pick and place environment to test the agent’s capability to generalise over different surgical scenes. The box-plot, in Fig. 6, shows that despite the initial distance between target and robot, the agent can correctly generalise and always solve the task complying with the acceptance threshold. The presented results prove that the agent trained with the randomisation of different environment variables can generalise over unseen volumes and environment meta data equally. We aim to translate all the skills learned in simulation to the real robot, especially testing how the generalisation capabilities can be transferred from sim-to-real. Adjustments can be done also in the simulation environment to improve training performance when all the environment variables are randomised, improving, for example, randomisation conditions and maximum amount of time steps, as well as more detailed characterization of the interaction between obstacles and successful grasping. Further work will involve a better comparison with the benchmark environment focusing on the differences between environment variables. Moreover, clinical experiments will be carried out to better assess the clinical accuracy required during the intraoperative organ repositioning. Promising future extensions of *rlman* involve the implementation of different skills allowing the agent to transfer-learn across them and extending the high-dimensional states to multiple tasks. In this scenario, the agent would be able to select which task to execute based on the type of state information that will receive as input.

## Supplementary Information

Below is the link to the electronic supplementary material.

Supplementary file 1 (pdf 7952 KB)
