# Supplementary material for: Learning intraoperative organ manipulation with context-based reinforcement learning
Source: Int J Comput Assist Radiol Surg. 2022 May 3;17(8):1419–27. doi: 10.1007/s11548-022-02630-2 (PMC9307544; doi:10.1007/s11548-022-02630-2)
Supplement: Supplementary file 1 — (pdf 7952 KB) [file 11548_2022_2630_MOESM1_ESM.pdf]

Learning Intraoperative Organ Manipulation with Context-based Reinforcement Learning

Claudia D'Ettorre[1*], Silvia Zirino[1,2], Neri Niccolò Dei[3], Agostino Stilli[1], Elena De Momi[2] and Danail Stoyanov[1]

[1*]Wellcome / EPSRC Centre for International and Surgical Sciences (WEISS), University College London, London, UK.
[2] Department of Electronics, Information and Bioengineering (NearLab), Politecnico of Milan, Milan, Italy.
[3] The BioRobotics Institute, Scuola Superiore Sant'Anna, Italy.

*Corresponding author(s). E-mail(s): c.dettorre@ucl.ac.uk;

1 Pneumatic Attachable Flexible Rail

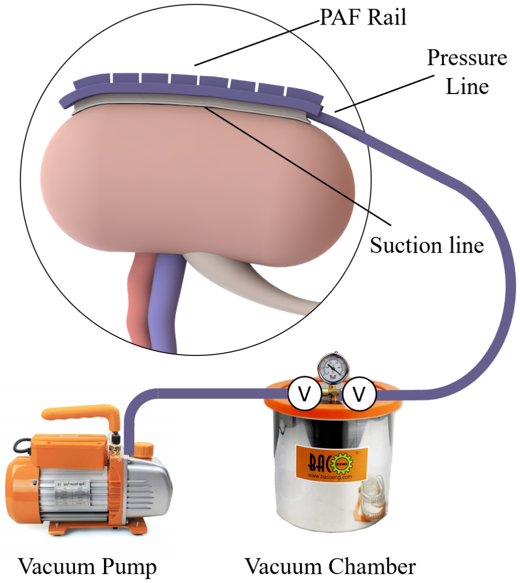

Fig. 1: Model of the PAF rail deployed on the kidney surface. The system can be attached to tissues and organ surfaces thanks to the vacuum pressure in the suction cups (gray portion of the system in the figure). A pump creates vacuum in an air-tight chamber which is connected to the system via the pressure line. Typically large organs, such as the liver, are manipulated with dedicated retractors, which are normally held by an assistant through the support trocar port. For smaller organs or portions of tissues, the main surgeon operates one of the three arms to manipulate and retract smaller organs like the kidney as well as portions of the bowels in the desired position, actively controlling one arm at the time, positioning it to hold the targeted organ/tissue in the desired position and locking it in place, moving to the next arm if needed and repeating the sequence. Besides the standard laparoscopic retractors, surgeons often also use the shaft of the robotic tools to manipulate the organ towards the desired position. This is a challenging procedure especially due to the geometry of the shaft that requires the surgeon to continuously reposition the tool until the desired position is reached. The PAF rail has been designed with the idea of simplifying these steps, significantly reducing the risk of damages associated with the interaction of rigid tools with soft tissues.

2 Kidney phantom

An anatomically detailed kidney model was added to the scene to simulate an operative field. It was resized to fit a realistic bounding box of 14x8.1x5.4 (length, width, height) cm^3. A texture was overlaid using pictures of explanted porcine kidneys. The result is displayed in figure 2a. To accelerate computation in the simulation environment, the kidney was approximated using a cuboid whose dimensions neatly fit the organ model. The approximation error of the surface of the kidney was computed over fifteen equally spaced points positioned on the surface of the model and projected on the corresponding surface of the cuboid, resulting in under 2 mm of average distance. Figure 2b illustrates this setup, with the blue plane and white points belonging to the cuboid and kidney surface, respectively. Moreover, this approximation significantly simplifies the place task, laying the Rail on a plane surface. As shown in figure 2c. The kidney has five triplets of dummies on its top surface, randomly used as targets for the placement of the PAF Rail.

The environment has been developed to allow randomisation of the initial positions of the objects, guaranteeing stochasticity. The randomisation volumes are normalised according to a fixed initial position of the robot, which can itself have a random pose at reset. The randomisation of the kidney is performed following these steps:

1. The horizontal-plane x and y coordinates of the centre of mass of the cuboids are sampled in the interval [-50, 50] mm;
2. The height is lowered with the addition of a vertical translation, given by the projection of the initial position of the robot (fixed vector) on the operative table;
3. The height of the centre of mass is set to 38 mm off the table: this height avoids penetration of the table at reset, whatever the orientation of the kidney;
4. The orientation of the cuboid is randomised in the following intervals, which were chosen to allow some randomisation, yet avoiding too oblique orientations:
   - Roll angle between [-30°, 30°]
   - Pitch angle between [-20°, 20°]
   - Yaw angle between [-90°, 0°]

The random orientation allows the targets to have random heights. The kidney, therefore, is mid-air in some circumstances. This was not considered an issue because the operative field simulates an abdominal cavity, where the kidney is not always flat on a plane surface nor has a portion always in contact with a specific horizontal plane. Given the previous dimensions, the robot is allowed to reach any point in a cube of side 17 cm

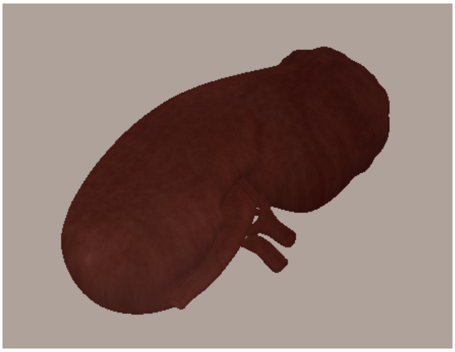

(a)

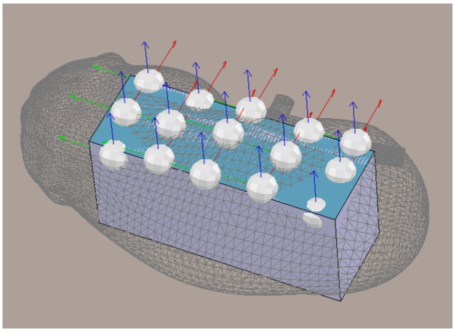

(b)

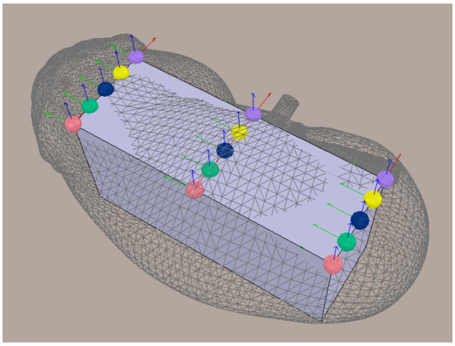

(c)

Fig. 2: (a):Kidney model. (b):Setup to measure the kidney surface approximation error. (c): Target dummies on the kidney's cuboid: triplets of central, top and bottom dummies (same color) define the place targets.

3 Clinical Accuracy

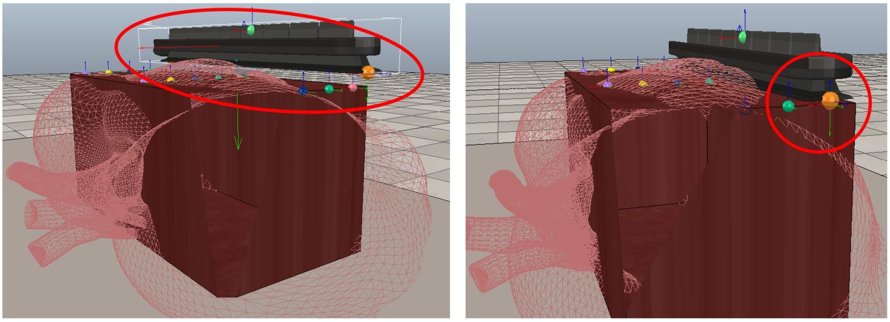

Fig. 3: As mentioned in Section 1, the PAF rail is able to correctly engaged with the organ when the surface of the suction line is parallel to the organ surface. Experiments have been carried out with explanted porcine kidneys and liver showing that this is the only configuration that permits active suction.

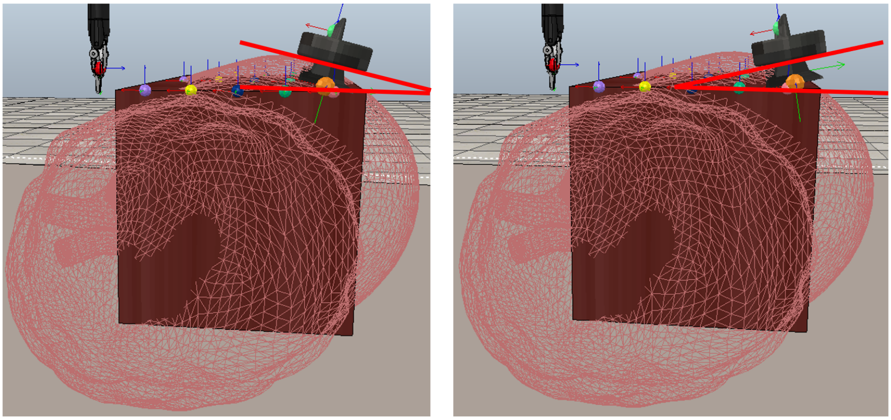

Fig. 4: Snapshot of two wrong configurations. The error in orientation is measured computing the distance between the reference frame of the dummy over the kidney and the one on the bottom of the PAF rail.

4 Reaching Target Task

The Reaching task has been tested in a 3D environment where the initial target position is randomised, while the tooltip initial pose is fixed.

4.1 Agent

For this task, the agent is represented by the DQN deep neural network built according to the original one described by Mnih *et al.* in "Human-Level Control through Deep Reinforcement Learning", and whose architecture details can be found below.

Convolutional Neural Network Architecture: The convolutional neural network employed for the Deep Q-Network is from the paper by Mnih *et al.*, except for the input image dimension that has been decreased to reduce the computational demand.

Input dimension to neural network: *64 x 64 x 4*
First hidden layer: *convolutional*

- number of filters: *32*
- kernel size: *8*
- stride: *4*
- rectifier non linearity (ReLU)

Second hidden layer: *convolutional*

- number of filters: *64*
- kernel size: *4*
- stride: *2*
- rectifier non linearity (ReLU)

Third hidden layer: *convolutional*

- number of filters: *64*
- kernel size: *3*
- stride: *1*
- rectifier non linearity (ReLU)

Final hidden layer: *fully-connected*

- rectifier units: *512*

4.2 State Observation

The observation for this task is the image acquired by a single vision sensor attached to an Endoscope Camera Manipulator. Specifically, this sensor can render the objects present in its field of view, and the content of the images that it captures is accessible through the API of the employed simulator. The frames that are actually inputted to the agent are preprocessed by first converting them from RGB to grayscale and by finally down-sampling them to 64x64

images to decrease the computational load (see Figure 5). The preprocessing step is applied to the four most recent frames stacked on the width dimension to produce the deep neural network input (i.e. 64x64x4 image). This is done to provide the network with state information over time (i.e. the arm direction of motion and velocity cannot be determined from a single frame), which is fundamental for the agent to perform well in environments with movement.

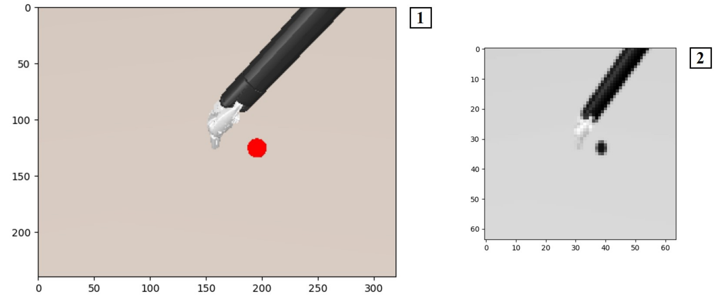

Fig. 5: Input images from Reaching Environment: **1 -** original RGB 320x240 image. **2 -** preprocessed greyscale 64x64 image.

4.3 Action

In order to employ the Deep Q-Network DRL algorithm, the action space has to be discrete. In this environment, the agent can choose among seven possible actions at each time-step: the arm can stay still or move forward and backwards by 3mm in the three spatial dimensions within a predefined cubic area of 30x30x30 mm inside the vision sensor field of view.

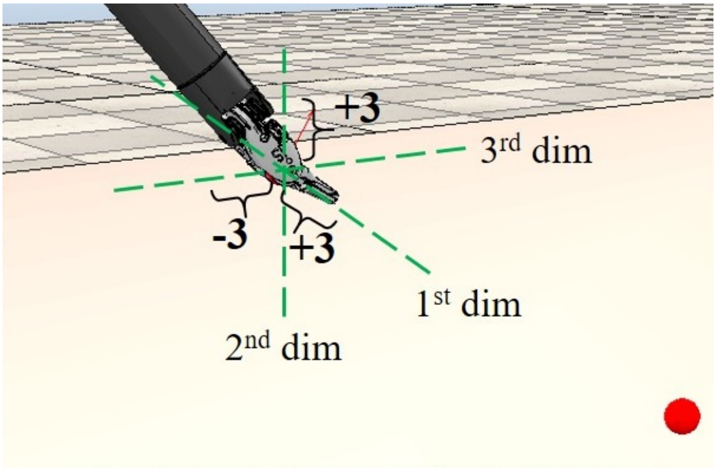

Fig. 6: Discrete Actions: movement directions (dashed green lines) and step sizes for the Reaching Task.

5 Pick and Place meta data environment

refer to section 4.3 adding a picture of the environment

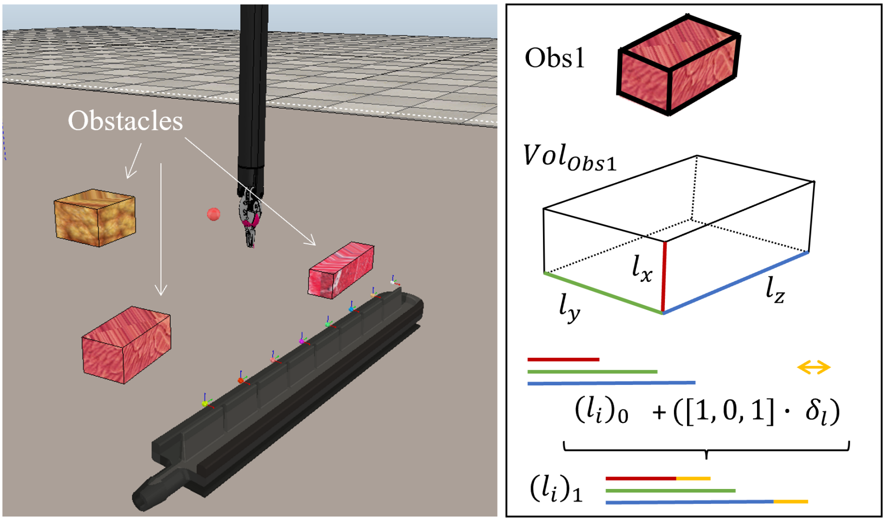

Fig. 7: Representation of the PickPlaceRail environment augmented with obstacles. On the right schematic on how the dimension is modified at each reset.

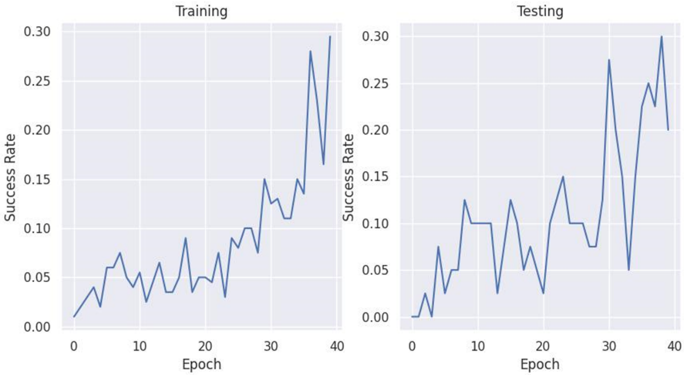

Fig. 8: Diagram of the training and testing success rate of the PPR task with meta data.

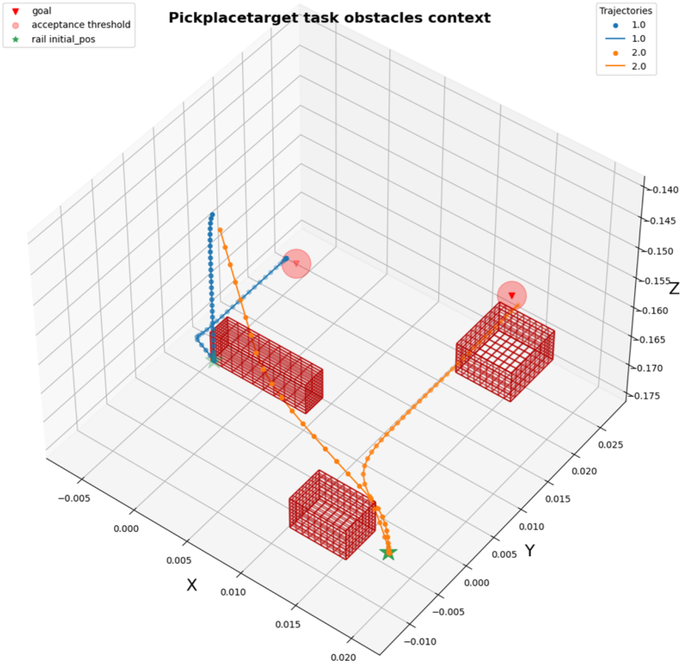

Fig. 9: Representation of two episode's trajectory of the PPR task. The green star represents the grasp site of the rail, the red triangle the target, the red shaded area the acceptance threshold. The obstacle are defined by the red meshes and the trajectory are highlighted in blue and orange.

6 Skills Benchmark

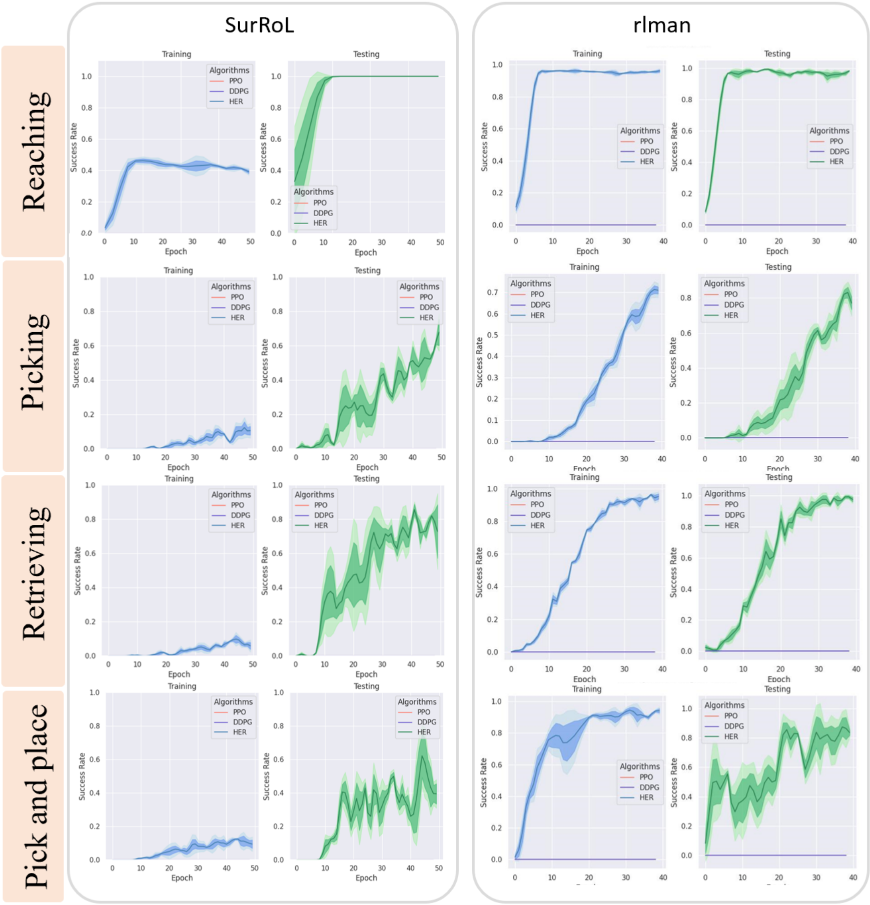

Fig. 10: Complete representation of the results of the evaluation of the analysed skills for each environment and comparison with the SurRoL benchmarks, for training and testing.

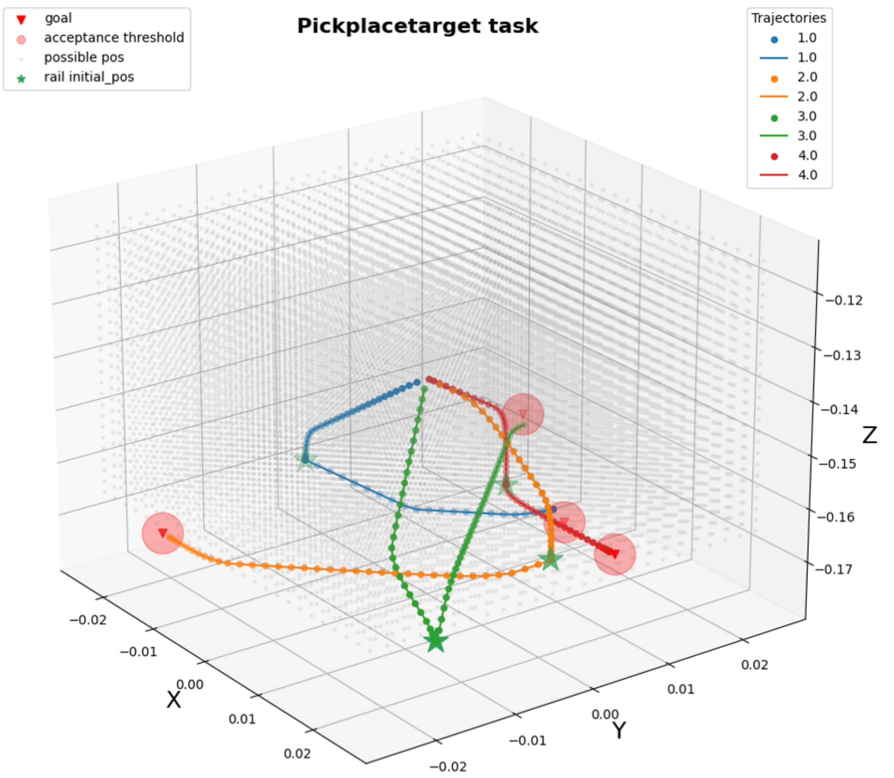

Fig. 11: Sample of four episodes' trajectories. The green star represents the rail's grasping site, the red triangle the target, the red shaded area the acceptance threshold and the robot's trajectories are highlighted in the different colors.

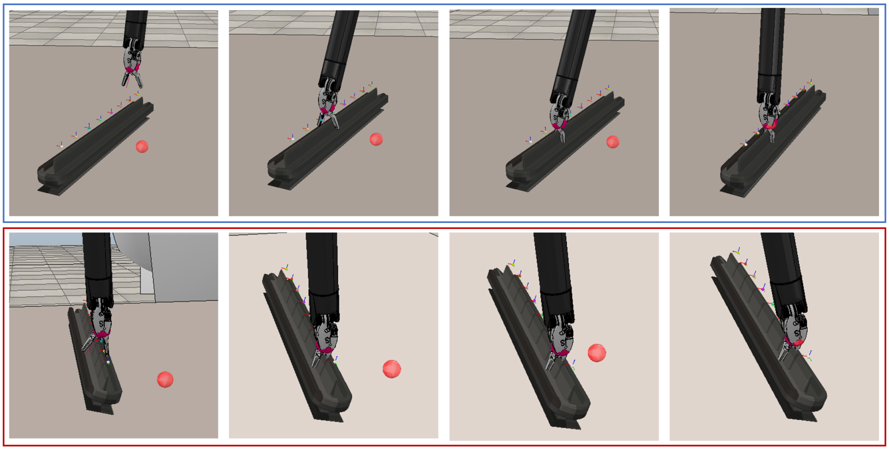

Fig. 12: Sample of environment captured at different time steps for two different trajectories execution (blue and red).

7 Pick and Place results: generalisation experiments

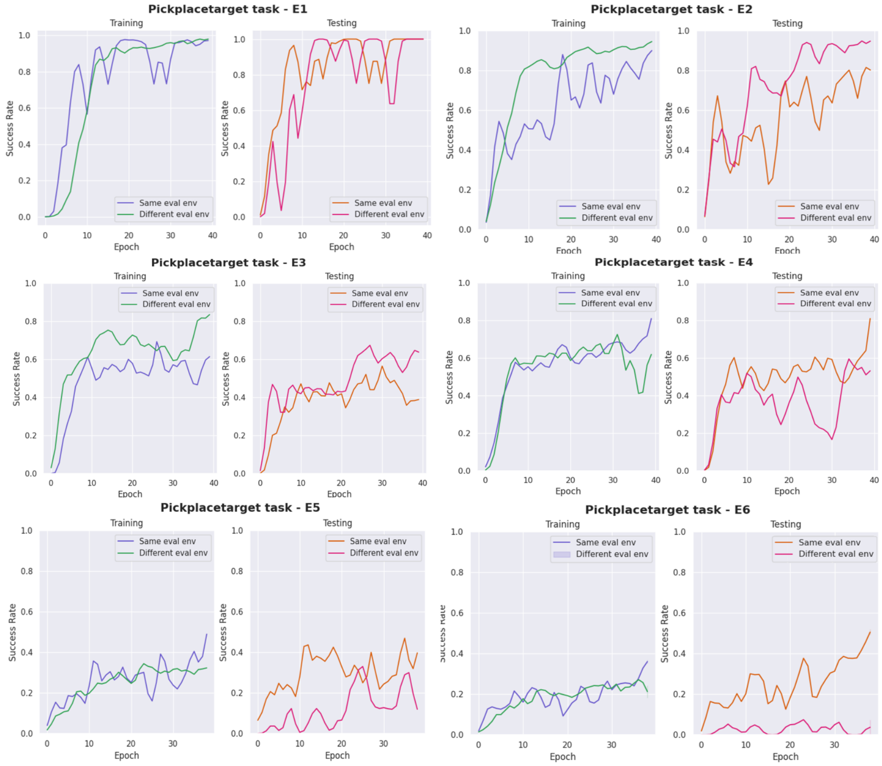

Fig. 13: Complete representation of the generalisation experiments over the environment variables.

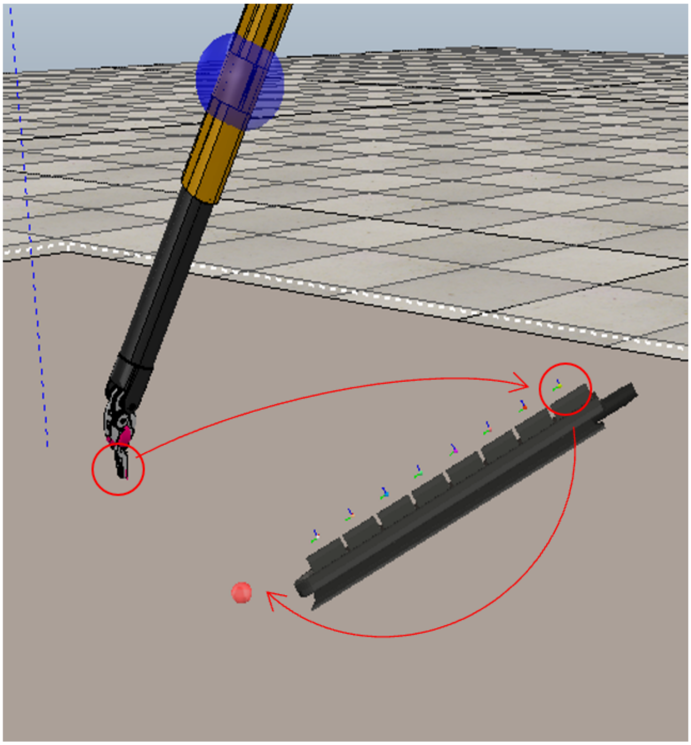

Fig. 14: Example of a episode's failure. The relative distance between tool, rail and target is too large to be travel within the maximum time steps.

Declarations

Funding. This research was funded in whole, or in part, by the Wellcome/EPSRC Centre for Interventional and Surgical Sciences (WEISS) [203145/Z/16/Z]; the Engineering and Physical Sciences Research Council (EPSRC) [EP/P027938/1, EP/R004080/1, EP/P012841/1]; and the Royal Academy of Engineering Chair in Emerging Technologies Scheme. For the purpose of open access, the author has applied a CC BY public copyright licence to any author accepted manuscript version arising from this submission.

Conflict of interest. The authors declare that they have no conflict of interest.

Ethical approval. This article does not contain any studies with human participants or animals performed by any of the authors.

Informed consent. This articles does not contain patient data
